# Phenolics and Polyphenolics from Melastomataceae Species

**DOI:** 10.3390/molecules201017818

**Published:** 2015-09-25

**Authors:** Diana Marcela Ocampo Serna, José Hipólito Isaza Martínez

**Affiliations:** 1 Grupo de Investigación en Productos Naturales y Alimentos (GIPNA), Departamento de Química, Facultad de Ciencias Naturales y Exactas, Universidad del Valle, Edificio 320, Oficina 2096, Ciudad Universitaria-Meléndez, Calle 13 No. 100-00, Cali 760032, Colombia; E-Mail: diana.ocampo@ucaldas.edu.co; 2 Departamento de Química, Facultad de Ciencias Exactas y Naturales, Universidad de Caldas, Calle 65 No. 26-10, Manizales 170004, Colombia

**Keywords:** melastomataceae, flavonoids, polyphenols, hydrolizable tannins, biogenesis, nobotannins, melastoflorins, anthocyanins

## Abstract

The Melastomataceae family, the seventh largest flowering plants, has been studied in several fronts of natural product chemistry, including terpenoids, simple phenolics, flavonoids, quinones, lignans and their glycosides, as well as a vast range of tannins or polyphenols. This review concerns the phenolic and polyphenolic metabolites described in the literature for several genera of this family, the mode of isolation and purification, and the structure elucidation of these new natural products that has been achieved by extensive spectral analyses, including ESI-MS, ^1^H-, ^13^C-NMR spectra and two-dimensional experiments, COSY, TOCSY, *J*-resolved, NOESY, HMQC, DEPT, and HMBC, as well as chemical and enzymatic degradations and the chemotaxonomic meaning. Finally, a general biogenetic pathway map for ellagitannins is proposed on the bases of the most plausible free radical C-O oxidative coupling.

## 1. Introduction

The Melastomataceae Jussieu is a large family of *ca.* 166 genera and 4200–4500 species that exhibit a diversity of growth habitats, including herbs, shrubs, treelets, climbers, and trees up to 30–45 m high; the last are concentrated in the Merianieae and Miconieae tribes. They can be easily recognized among dicotyledoneous by their leaves which have a characteristic acrodromous venation. Although distributed pantropically, the family has a marked concentration of species in the New World where there are *ca.* 2950 species, while about 1275–1550 are found in the Old World [1,2].

Due to the taxonomic complexity, researchers continue to investigate its phylogenetic and classification today [3,4,5,6,7]. It has been scarcely investigated from a phytochemical research viewpoint. Although a large number of compounds have been isolated, only a few species have been extensively studied. The major constituents of this family belong to terpenoids, simple phenolics, quinones, lignans and their glycosides, as well as a vast range of tannins or polyphenols, mainly hydrolyzable tannin oligomers of molecular weights up to 4600 Da, and some flavonoids and acylated anthocyanins. The tannins found in various plants of this group may be responsible for the traditional medicinal applications, especially in Asia and Latin America. This review concerns the phenolic and polyphenolic metabolites described in the literature for several genera of this family, the mode of isolation and purification, and the structure elucidation of these new natural products that has been achieved by extensive spectral analyses, including ESI-MS, ^1^H-, ^13^C-NMR spectra and two-dimensional (2D) experiments, COSY, TOCSY, *J*-resolved, NOESY, HSQC, DEPT, and HMBC, as well as chemical and enzymatic degradations. Also, the chemotaxonomic meaning ascribed to these compounds will be discussed. Finally, a general biogenetic pathway map for ellagitannins is proposed on the basis of the most plausible free radical C-O oxidative coupling. The taxonomy and folk medicinal uses of Melastomataceae plants are described in Table S1 in the Supplementary File.

## 2. Chemical Constituents of Melastomataceous Plants

Most of the reported chemical constituents from the Melastomataceous plants belong to polyphenolic compounds; however, some triterpenoids and alkyl benzoquinones [8,9,10,11] have also been described. Alkaloids have been detected but not isolated in *Clidemia hirta D.* Don and *Sonerila heterostemon* Naudin [12].

### 2.1. Triterpenoids and Alkyl Benzoquinones

**Figure 1:**
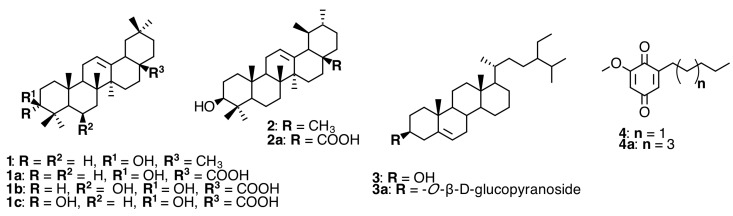
Triterpenoids, steroids, and alkyl benzoquinones.

Leaves of *Miconia stenostachya* DC. gave two triterpenoids, sumaresinolic acid (**1b**) and 3-epi-sumaresinolic acid (**1c**), from the ethanolic extract [8]. The plant *Miconia lepidota* DC. yielded the alkyl benzoquinones 2-methoxy-6-pentyl-3,5-cyclohexadiene-l,4-dione (**4**) and 2-methoxy-6-heptyl-3,5-cyclohexadiene-1,4-dione (**4a**) as bioactive compounds [9]. Compounds obtained from *Dissotis perkinsiae* Gilg were identified as oleanolic acid (**1a**), ursolic acid (**2a**), and 3-*O*-β-d-glucopyranoside of sitosterol (**3a**) [10]. The *T. candolleana* (Mart. ex DC.) Cogn. *n-*hexane extract analyzed by HRGC led to identification of β-sitosterol (**3**), β-amirin (**1**), and α-amirin (**2**); **1a** and **2a** were also isolated from *T. candolleana* (Mart. ex DC.) Cogn. methylene chloride extract [11] (see Figure 1 and Table S2 in Supplementary File).

### 2.2. Flavonoids

**Figure 2:**
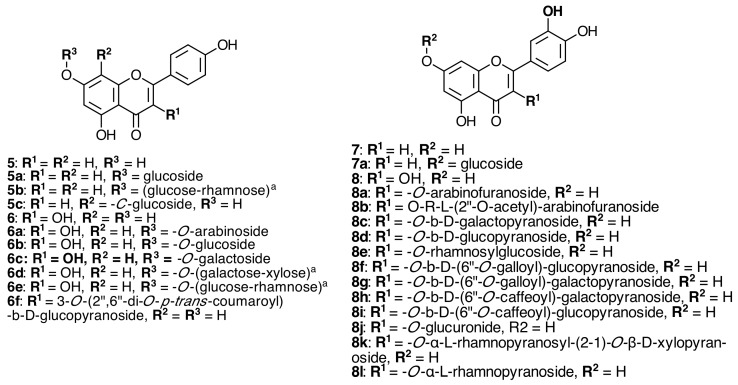
Apigenin, kaempferol, luteolin and quercetin derivatives isolated from Melastomataceae species.

**Figure 3:**
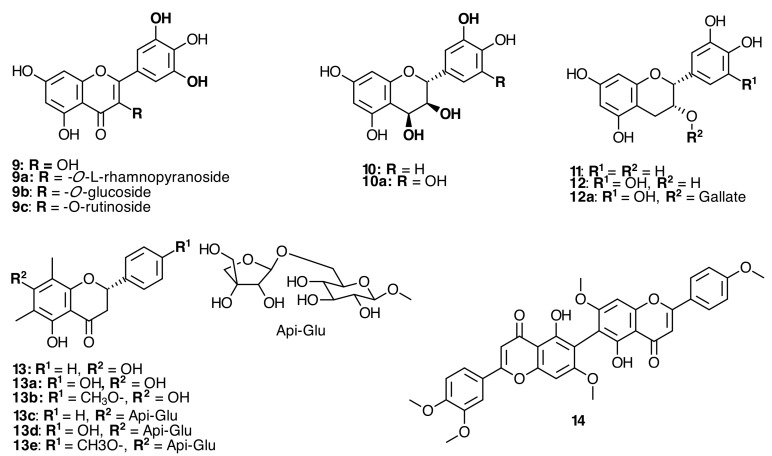
Flavonoids and derivatives isolated from Melastomataceae species.

**Figure 4:**
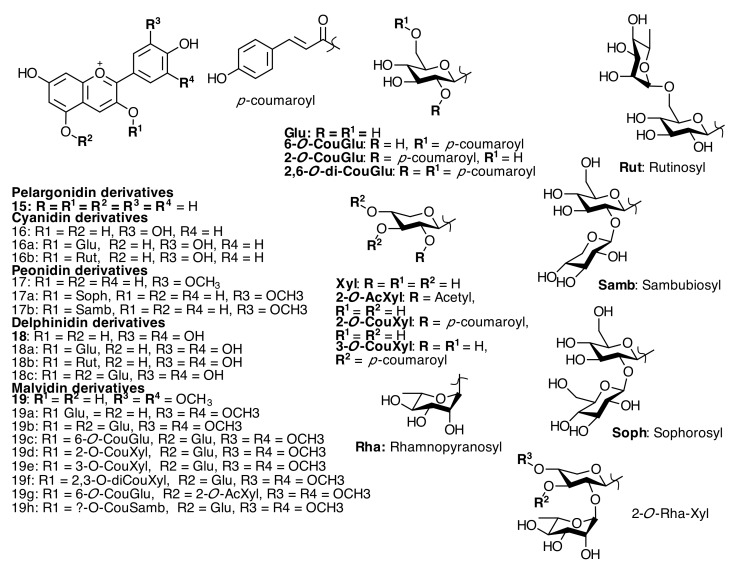
Anthocyanins and anthocyanidins isolated from Melastomataceae species.

In 1928, Molisch first described the presence of anthocyanins in the adventitious root hairs of some melastomataceae species, such as *Centradenia grandiflora* Endl Ex Walp, *Monochaetum umbellatum* Naudin, *Tibouchina semidecandra* Cogn., *Medinilla magnifica* Lindley, *Bertolonia aenea* Naudin, *B. marmorata* Naudin, and *B. vittata* L. [12,13]. Later, he reported the isolation of various kinds of flavonoids from this plant family. Kaempferol (**6**) was found in *M. magnifica* Lindley and *Centradenia floribunda* Planch. *Bertolonia marmorata* Naudin, *Medinilla magnifica* Lindley, and *Tibouchina ciliaris* (vent.) Cong. contain quercetin (**8**), leucocyanidin (**10**), and leucodelphinidin (**10a**) [13,14] (see Figure 2, Figure 3 and Figure 4).

Five known flavonol glycosides were also identified from *Monochaetum multiflorum* (Bonpl.) Naudin by comparison of their UV-vis and ^1^H-NMR spectra with those reported in the literature as isoquercitrin (**8d**), hyperin (**8c**) [15], trifolin (**6c**) [16], quercetin 3-(6ʹʹ-*O*-caffeoyl)-β-d-glucopyranoside (**8i**) [17], and 3-(6ʹʹ-*O*-caffeoyl)-β-d-galactopyranoside (**8h**) (Figure 2) [18]. Compounds **8d**, **8c**, **8h**, **8i**, and **6c** were reported for the first time in Melastomataceae. Table S3 in the Supplementary File and Figure 2, Figure 3 and Figure 4 show that flavonoids, flavonoid glycosides, and acyl glycosides could be useful as chemotaxonomic markers at generic and specific levels in Melastomataceous plants. Hyperin (**8c**) and isoquercitrin (**8d**) were the most abundant constituents in the EtOAc extract and in the whole aqueous acetone homogenate of *M. multiflorum* (Bonpl.) Naudin [19]. The acyl glycosides **8i** and **8h** could be effective anti-complement agents that would be useful as anti-inflammatory medicines owing to the sequence flavonol-sugar-aromatic side chain, which was demonstrated to be essential for potent anti-complement activity of related compounds isolated from *Persicaria lapathifolia* L. [20].

The evaluation of the taxonomic value of leaf flavonoids from eight species of *Huberia* from the Atlantic forest led to the isolation and identification of 17 compound derivatives from the aglycones apigenin (**5**), luteolin (**7**), kaempferol (**6**), and quercetin (**8**) [21]. *Dissotis perkinsiae* Gilg contains quercetin 3-*O*-(6ʹʹ-*O*-galloyl)-β-galactopyranoside (**8g**) (Figure 2) [10].

Butanol extract of *Miconia rubiginosa* Bonpl. DC. [22] and *Clidemia rubra* (Aubl.) Mart. [23] led to the isolation of catechin (**11**), epicatechin (**12**), and epigallocatechin gallate (**12a**) (Figure 3). The methanolic extract of *Miconia prasina* (Sw.) DC. stems yielded a glycosidic flavanone and miconioside C (**13d**), along with 7-*O-*β-d-apiofuranosyl-(1-6)-β-d-glucopyranosyl matteucinol (**13d**), miconioside B (**13e**), matteucinol (**13a**), farrerol (**13b**), and desmethoxymatteucinol (**13c**) (Figure 3) [24].

The analysis of leaves of *Lavoisiera*, *Microlicia*, and *Trembleya* species gave a total of 116 compounds, comprising 69 flavonols and 47 flavone glycosides. *Lavoisiera* is characterized by 6-oxygenated derivatives, while flavonols predominate in *Microlicia* species [25].

**Figure 5:**
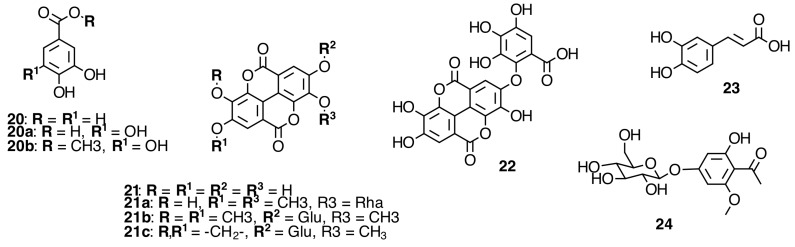
Phenolic acids and glycosyl derivatives isolated from melastomataceae species.

The first example of a C_6_–C_6_ʹʹ linked flavone dimer, 5-hydroxy-4ʹ,7-dimethoxyflavone-(6-C-6ʹʹ)-5ʹʹ-hydroxy-3ʹʹʹ,4ʹʹʹ,7ʹʹ-trimethoxyflavone (**14**), was isolated from *Miconia cabucu* Hoehne and *M. rubiginosa* (Bonpl.) DC. (see Figure 3) methanolic extracts of the leaves along with the known compounds quercetin-3-*O*-α-l-rhamnopyranosyl-(2→1)-*O*-β-d-xylopyranoside (**8k**), quercetin-3-*O*-α-l-rhamnopyranoside (**8f**), myricetin-3-*O*-α-l-rhamnopyranoside (**9a**), myricetin 3-*O*-rhamnoside (**9c**), quercetin-3-*O*-β-d-glucopyranoside (**8d**), kaempferol-3-*O*-β-d-(2ʹʹ,6ʹʹ-di-*O*-*trans*-coumaroyl)-glucopyranoside (**6e**) (Figure 2), and gallic acid (**20a**) (Figure 5) [26]. In *Clidemia rubra* (Aubl.) Mart., the presence of a myricetin aglycone confirmed the identification of myricetin 3-*O*-glucoside (**9b**) and myricetin 3-*O*-rhamnoside (**9c**) [23].

From *T. semidecandra* (Mart. and Schrank ex DC.) Cogn. quercetin (**8**), myricetin (**9**), leucocyanidin (**10**), leucodelphinidin (**10a**) [27], quercetin 3-*O*-α-l-arabinofuranoside (avicularin) (**8a**), and hyperin 6ʹʹ-*O*-gallate (**8g**) were isolated [28]. lsoquercitrin 6ʹʹ-*O*-gallate (**8f**) was obtained from the leaves of *Melastoma malabathricum* L. [29] and *Dissotis perkinsiae* Gilg [10] and myricitrin (**9a**) (Figure 2) from *M. normale* D. Don [30]. A *C*-glucosyl flavone, vitexin (**5c**) (Figure 2), was also isolated from *Melastoma* *dodecandrum* Lour [31].

Luteolin (**7**) and the isoflavone genistein have been reported from *T. candolleana* (Mart. ex DC.) Cogn. ethanol extract [11]. Quercetin (**8**), quercetin 3-*O*-α-l-(2ʹʹ-*O*-acetyl)-arabinofuranoside (**8b**), and quercitrin (**8e**) were isolated from *T. Semidecandra* (Mart. and Schrank ex DC.) Cogn. and were potential free radical scavengers and promising natural tyrosinase inhibitors [32].

### 2.3. Anthocyanins and Anthocyanidins

All anthocyanins isolated from Melastomataceous plants (see Figure 4 and Table S4 in the Supplementary File) are pelargonidin (**15**), cyanidin (**16**), peonidin (**17**), delphinidin (**18**), and malvidin (**19**) glycosides or acyl glycosides. Malvin (**19b**) (malvidin 3,5-diglucoside) and tibouchinin (**19c**) [malvidin 3-(6ʹʹ-*p*-coumaroylglucosyl)-5-glucoside] occur in *Melastoma malabathricum* L. and *T. semidecandra* (Mart. and Schrank ex DC.) Cogn., respectively [27,33,34]. Malvidin 3-*p*-coumaroylxylosyl-5-glucoside (**19d**), malvidin 3-(di-*p*-coumaroylxylosyl)-5-glucoside (**l9f**) [35], and malvidin 3-*p*­coumaroylsambubiosyl)-5-glucoside (**19h**) [36] were reported in *T. granulose* (Desr) Cogn., but the precise location of acyl groups was not clearly established. The major pigment found in the flowers of *T. urvilleana* Cong. was identified as 3-*O*-[6-O-(E)-p-coumaroyl-β-d-glucopyranosyl]-5-*O*-(2-*O*-acetyl-β-d-xylopyranosyl) malvidin (**19g**) [37]. The contents of acylated anthocyanins in the flowers of Melastomataceae give them economical importance as sources of natural food colorants.

Four anthocyanins from *Clidemia rubra* (Aubl.) Mart. berries were detected and identified by comparison of fragmentation pattern and retention time with authentic standards of delphinidin 3-*O*-glucoside (**18a**), delphinidin 3,5-*O*-diglucoside (**18c**), cyanidin 3-*O*-glucoside (**16a**), and cyanidin 3-*O*-rutinoside (**16b**) and a peak tentatively assigned to delphinidin 3-*O*-rutinoside (**18b**) showing a molecular ion [M + H]^+^ at *m*/*z* 611 [23]. A report of anthocyanins and anthocyanidins in different Melastomataceae varieties showed the presence of malvidin glycosides in the flowers (**19d**, **19e**, **19f**) and mainly delphinidin and pelargonidin glycosides in the fruits. An acylated delphinidin 3,5-*O*-diglucoside was found in *Clidemia hirta* Don [23].

Four pigments were extracted from the flowers of *Tibouchina grandiflora* and two of them were identified as peonidin-3-sophoroside (**17a**) and malvidin-3,5-diglucoside (**19b**). Another two pigments were tentatively identified as Malvidin-3-*O*-glucoside (**19a**) malvidin-3-(*p*-coumaroyl)-sambubioside-5-glucoside (**19h**), and peonidin-3-sambubioside (**17b**) [36]. All anthocyanins are summarized in Table S4 in the Supplementary File.

The suitable storage condition for colored anthocyanin pigments is in acidic solution (pH 0.5 and 1.0) kept in the dark and at a low temperature (4 °C) [38]. The anthocyanin pigments are stable in the dark at 4 °C for 26 days [39]. Dye-sensitized solar cells (DSSCs) had been fabricated with *Melastoma malabathricum* L. ethanol or de-ionized water fruit extracts as sensitizer, giving better results than the last one [40].

### 2.4. Phenolic Acids and Derivatives

Some phenolic acids and glycosyl derivatives have been isolated from Melastomataceae species (see Figure 5 and Table S5 in the Supplementary File). Caffeic acid (**23**) was observed in *Clidemia floribunda* Planch [41]. Ellagic acid (**21**) has been isolated from *Dissotis perkinsiae* Gilg [10] and 3,3ʹ-*O*-dimethyl ellagic acid 4-*O*-α-l-rhamnopyranoside (**21a**) was isolated from *T. Semidecandra* (Mart. and Schrank ex DC.) Cogn. [32]. From leaves of *Phyllagathis rotundifolia* (Jack) Bl. 3,3ʹ,4-tri-*O*-methylellagic acid 4′-*O*-β-d-glucopyranoside (**21b**) and 3′-*O*-methyl-3,4-methylenedioxyellagic acid 4′-*O*-β-d-glucopyranoside (**21c**), which produced the deprotonated parent ion [M − H]^−^ at *m*/*z* 505 and 489, respectively, in ESI-MS^n^ negative mode [42], were identified.

### 2.5. Galloylated Cyanogenic Glucosides and Benzyl Glycosides

**Figure 6:**
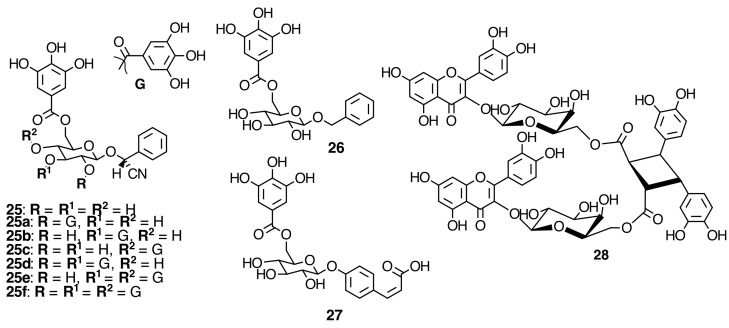
Galloylated cyanogenic glucosides and benzyl glycosides and di-hyperin ester of tetrahydroxy-µ-truxinic acid (monochaetin) from Melastomataceae species.

The EtOAc extract form *Monochaetum multiflorum* (Bonpl.) Naudin was submitted to a combination of chromatography on Toyopearl HW-40C, MCI Gel CHP-20P and/or YMC Gel ODS to yield three new galloyl glycosides, 6ʹ-*O*-galloylprunasin (**25**), benzyl 6ʹ-*O*-galloyl-β-d-glucopyranoside (**26**), and 4-*O*-(6ʹ-*O*-galloyl-β-d-glucopyranosyl)-*cis*-*p*-coumaric acid (**27**), and a novel di-hyperin ester of tetrahydroxy-µ-truxinic acid (monochaetin) (**28**), along with 26 known compounds (see Figure 6 and Table S6 in the Supplementary File). Among the known compounds, six were identified as simple phenols, gallic acid (**20a**), methyl gallate (**20b**) [35], protocatechuic acid (**20**), ellagic acid (**21**) [36,37], valoneic acid dilactone (**22**), and 4-*O*-β-d-glucopyranosyl-2-*O*-methylphloroacetophenone (**24**) [19]. The isolated compounds from *Monochaetum multiflorum* (Bonpl.) Naudin EtOAc extract were characterized based on the spectral analysis, and chemical or enzymatic degradation [19].

The compounds isolated from the leaves of *P. rotundifolia* (Jack) Bl. (see Figure 6 and Table S6 in the Supplementary File) and characterized by ESI-MS^n^ in negative ionization mode, four galloylated cyanogenic glucosides, were found to have similar fragment ions for the isomeric compounds such as prunasin digallate (**25a**–**c**) and prunasin trigallate (**25d**,**e**) [42]. The cyanogenic glucosides were identified by ESI-MS^n^ in negative mode as prunasin 6′-*O*-gallate (**25**), prunasin 2′,6′-di-*O*-gallate (**25a**), prunasin 3′,6′-di-*O*-gallate (**25b**), prunasin 4′,6′-di-*O*-gallate (**25c**), prunasin 2′,3′,6′-tri-*O*-gallate (**25d**), prunasin 3′,4′,6′-tri-*O*-gallate (**25e**), and prunasin 2′,3′,4′,6′-tetra-*O*-gallate (**25f**) [19,43,44]. Also, positive ionization mode has been used to identify cyanogenic glycosides [45].

### 2.6. Hydrolyzable Tannins

The major constituents of Melastomataceous plants belong to the hydrolyzable tannins. The monomeric hydrolyzable tannins hitherto isolated from Melastomataceous species are represented by the gallotannin and ellagitannin groups [46,47].

#### 2.6.1. Galloyl Glycosides

**Figure 7:**
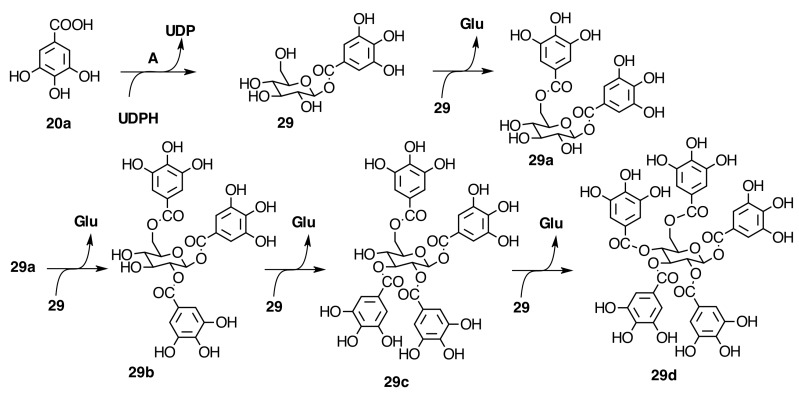
Biosynthesis of galloyl glucoses from gallic acid (**20a**) and uridin diphosphate glucose. A = UDP-glucose:gallate 1-*O*-galloyltransferase, UDP = uridine-5′-di-phosphate.

The gallotannins isolated from this family are 1,2,6-tri-*O*-galloyl-β-d-glucose (**29b**), 1,4,6-tri-*O*-galloyl-β-d-glucose (**31a**), and 1,2,3,6-tetra-*O*-galloyl-β-d-glucose (**29c**) (Figure 7) from EtOAc extract of fresh stems of *T. semidecandra* (Mart. and Schrank ex DC.) Cogn. [28]. Gallic acid 3-*O*-(6-*O*-galloyl)-β-d­glucopyranoside (**20c**) and methyl gallate (**20b**) were isolated from *Melastoma dodecandrum* Lour [31]. *M. malabathricum* L. [29] and *M. Normale* D. Don [30] have been reported to contain 1,4,6-tri-*O*-galloyl-β-d-glucose (**31a**) and 1,2,4,6-tetra-*O*-galloyl-β-d-glucose (**29l**) (see Figure 7 and Table S7 in the Supplementary File).

**Figure 8:**
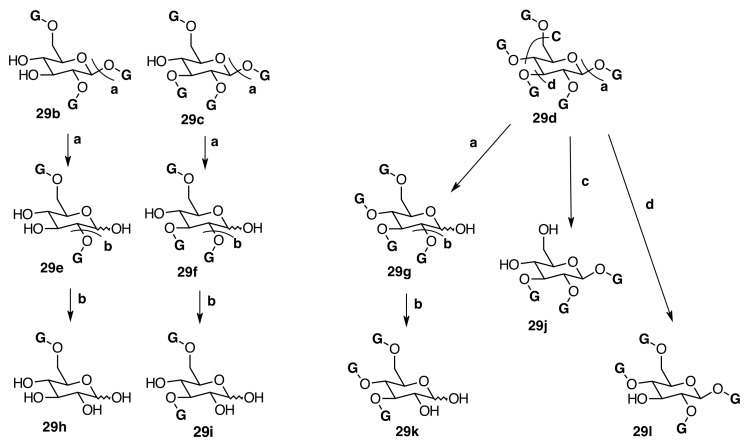
Degalloylation (**a**, **b**, **c**, **d**) of galloyl glucoses.

In *Phyllagathis rotundifolia* (Jack) Bl., seven gallotannins were identified as 6-*O*-galloyl-d-glucose (**29h**) [48], 3,6-di-*O*-galloyl-d-glucose (**29i**) [49], 1,2,3-tri-*O*-galloyl-β-d-glucose (**29j**) [50,51], 1,4,6-tri-*O*-galloyl-β-d-glucose (**31a**) [51,52,53], 3,4,6-tri-*O*-galloyl-d-glucose (**29k**) [49,54,55,56], 1,2,3,6-tetra-*O*-galloyl-β-d-glucose (**29c**) [55,57], and 1,2,3,4,6-penta-*O*-galloyl-β-d-glucose (**29d**) [58] (Figure 7 and Figure 8) by comparing their spectroscopic data with literature values [42]. All these anomeric galloyl glucoses present the anomeric effect evidenced in HPLC with two peaks and in ^1^H-NMR and ^13^C-NMR by the α/β anomeric proton and carbon signals, which allow the calculation of the anomeric ratio. Some other signals are also duplicated.

**Figure 9:**
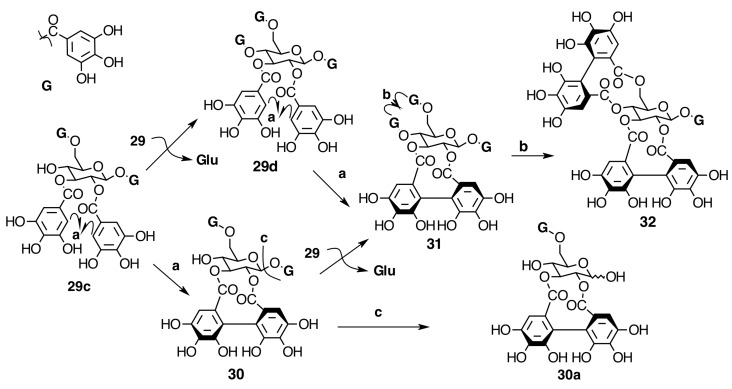
Biogenesis of initial monomeric precursors of oligomeric hydrolyzable tannins in Melastomataceae. Bold arrows indicate the pathway in Melastomataceae. **a**, **b** = C-C oxidative coupling by a free radical process. **c** = Degalloylation.

Gallotannins or galloylated esters of glucose are known to show elimination of several galloyl [M − H − 152]^−^ and gallate [M − H − 170]^−^ moieties in MS^n^ analysis [59,60,61]. The pentagalloyl glucose (**29d**) (Figure 9) (*m*/*z* 939) loses galloyl moieties to produce tetragalloyl glucose (*m*/*z* 787), trigalloyl glucose (*m*/*z* 635), digalloyl glucose (*m*/*z* 483), and monogalloyl glucose (*m*/*z* 331). The neutral mass loss of gallic acid [M − H − 170]^−^ moieties in pentagalloyl-glucose (*m*/*z* 939), tetragalloyl-glucose (*m*/*z* 787), trigalloyl-glucose (*m*/*z* 635), and digalloyl-glucose (*m*/*z* 483) are also observed. Elimination of the glucosyl moiety from monogalloyl-glucose was observed and subsequently formed the deprotonated gallic acid at *m*/*z* 169. The *m*/*z* 169 ion then underwent removal of CO_2_ to generate an ion at *m*/*z* 125 [42,61,62,63,64,65,66].

#### 2.6.2. Ellagitannins

Unlike the condensed tannins that are widespread throughout the plant kingdom, ellagitannins have been found only in dicotyledonous angiosperms. Among the plant families rich in ellagitannins are the Myrtaceae, Lythraceae, Onagraceae, Melastomataceae, and Combretaceae [67].

**Figure 10:**
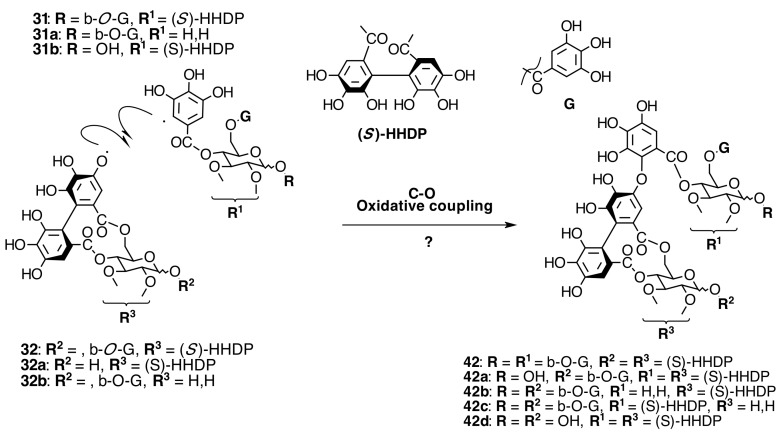
Route F in the biogenesis of dimeric ellagitannins in Melastomataceae. ? = Enzymes and reaction conditions are not yet clarified.

**Figure 11:**
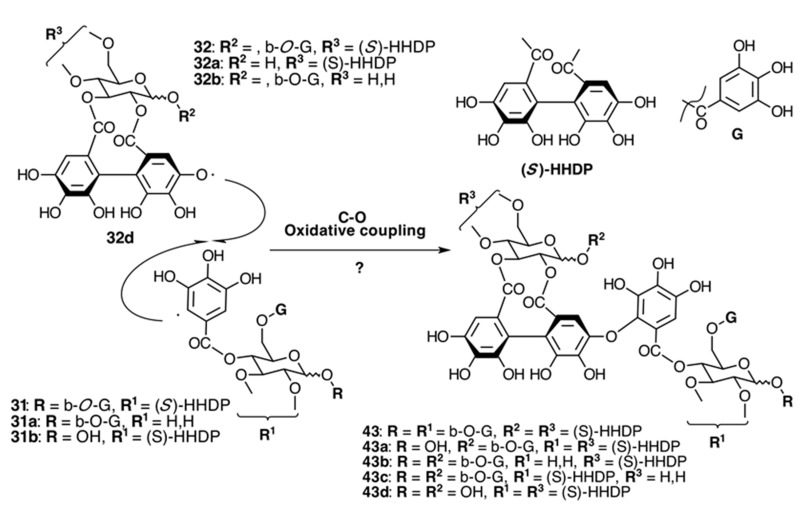
Route B in the biogenesis of dimeric ellagitannins in Melastomataceae. ? = Enzymes and reaction conditions are not yet clarified.

**Figure 12:**
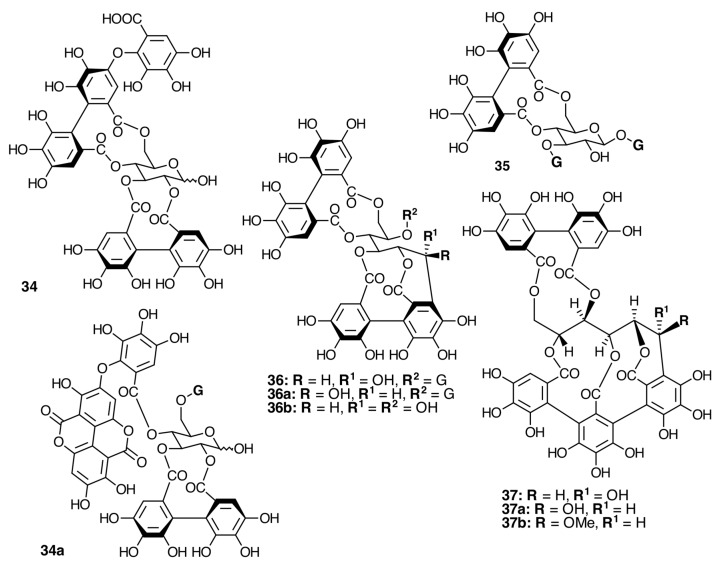
Modified and *C*-glucosidic monomeric ellagitannins.

**Figure 13:**
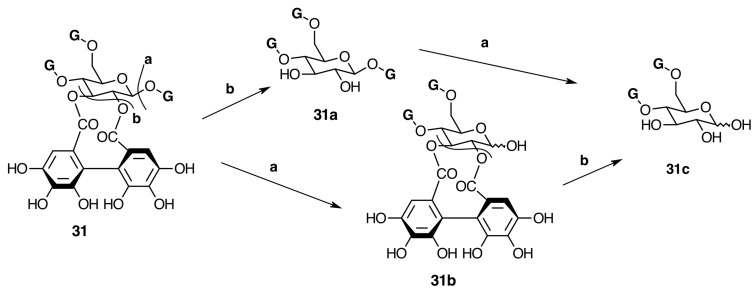
Degalloylation (**a**) and de-HHDP-ation (**b**, **c**) of pterocaryanin C (**31**).

**Figure 14:**
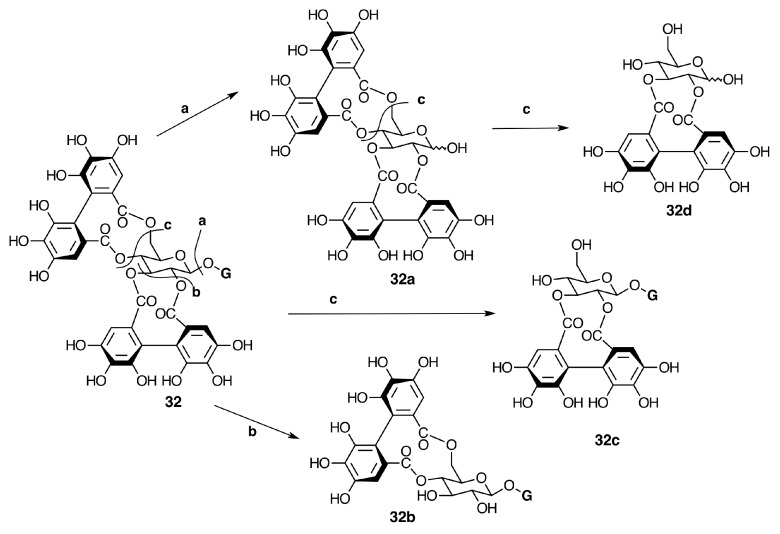
Biogenesis of casuarictin-type monomeric ellagitannins. Degalloylation (**a**) and de-HHDP-ation (**b**, **c**) of casuarictin (**32**).

**Figure 15:**
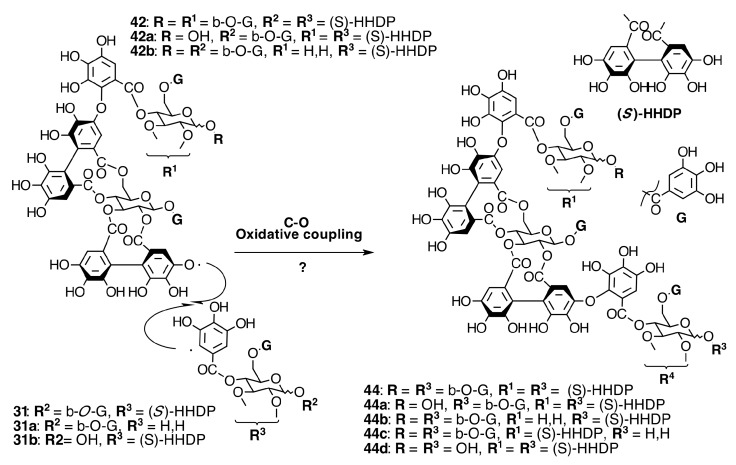
Route E in the biogenesis of trimeric ellagitannins in Melastomataceae. ? = Enzymes and reaction conditions are not yet clarified.

The occurrence of ellagitannins in this family was first suggested by the detection of ellagic acid (**21**) in *T. semidecandra* (Schrank and Mart. ex DC.) by chromatographic survey of the acid hydrolysate of the leaf extracts of various plant species [29,68]. It was demonstrated in 1986 with the isolation of a monomeric ellagitannin, nobotanin D (**30**) (Figure 9) and the dimers nobotanins A (**42a**), B (**43**), and F (**42**) (Figure 10 and Figure 11) from *T. semidecandra* (Schrank and Mart. ex DC.), along with a monomer, medinillin A (**34a**) (Figure 11) and a dimer, medinillin B (**42d**) (Figure 10) from *Medinilla magnifica* Lindl [69]. Next year, the structure of nobotanin B (**43**) was revised and fully characterized [70]. Continuing research on *T. semidecandra* (Schrank and Mart. ex DC.) led to the isolation of additional monomeric hydrolyzable tannins, casuarictin (**32**), pedunculagin (**32a**), praecoxin A (**34**) and B (**31b**), casuarinin (**36**), 2,3-*O*-(*S*)-hexahydroxydiphenoyl-d-glucopyranose (**32d**), and three *C*-glucosidic tannins identified as castalagin (**37**), vescalagin (**37a**), and 1-*O*-methylvescalagin (**37b**) (Figure 9, Figure 10, Figure 11, Figure 12, Figure 13 and Figure 14) [28]. Trimeric ellagitannins nobotanins E (**44**) and C (**44a**) (Figure 15) were isolated from the leaf extracts of the same plant and were fully characterized on the basis of chemical and spectral evidence [71].

#### 2.6.3. Biogenesis of Ellagitannins in Melastomataceous Plants

A previous description of ellagitannin biogenesis has been reported [72]; however, Melastomataceous species show some differences as depicted in the following paragraphs.

##### Biogenesis of Monomeric Hydrolyzable Tannins in Melastomataceae

**Figure 16:**
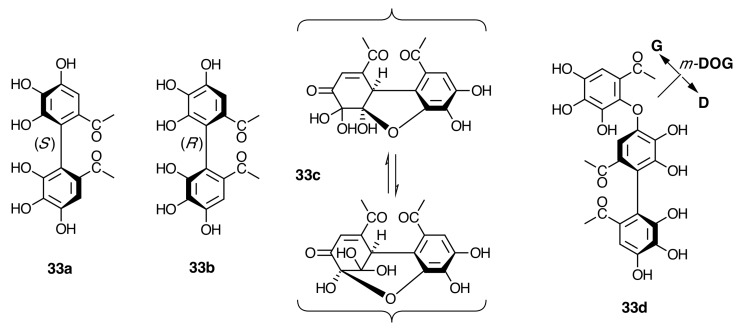
(*S*)-hexahydroxydiphenoyl (*S*)-HHDP (**33a**), (*R*)-HHDP (**33b**), dehydrohexahydroxydiphenoyl (*R*)-DHHDP (**33c**), and valoneoyl (**33d**) moieties of oligomeric ellagitannins.

The biogenesis of both classes of hydrolyzable tannins, gallotannins and ellagitannins, has been described, the former of which is produced by a stepwise galloylation of glucose that begins with the formation of β-glucogallin (**29**) (Figure 7), the first intermediate and principal activated acyl donor, from gallic acid and the ubiquitous uridin-5ʹ-diphosphateglucose (UDP-glucose) catalyzed by a glucosyl transferase [73]. A series of consecutive and highly regiospecific galloyl transfers between β-glucogallin (acts as both galloyl donor and acceptor) catalyzed by an acyltransferase, β-glucogallin: 1,2,3,4,6-pentagalloyl-β-d-glucose (3-*O*-galloyl)-galloyl transferase lead to 1,2,3,4,6-penta-*O*-galloyl-β-d-glucose (β-PGG) (Figure 7) [74]. The equilibration between both ^4^*C*_1_ (more stable) and ^1^*C*_4_ (less stable) chair conformations of β-PGG would branch the biosynthetic pathway of ellagitannins. The former leads to the (*S*)-HHDP (Figure 16) esters of glucose and the latter to the (*R*)-HHDP derivatives by C-C oxidative coupling of galloyl groups [75,76].

The first step in the aryl coupling sequence in the pathway from β-PGG to (*S*)-HHDP ellagitannins would start between 4,6-*O*-galloyl groups to produce tellimagrandin II and then between 2,3-*O*-galloyl groups yielding casuarictin (**32**) (Figure 9 and Figure 14). Degalloylation of these tannins produces tellimagrandin I and pedunculagin (**32a**), respectively (Figure 14). The formation of HHDP can be hypothesized as a free radical process involving enzymes like laccases or peroxidades with a lower redox potential than those associated with lignifications [76]. The stereospecific character of the HHDP group may be aided by the participation of an auxiliary (dirigent) protein as in the production of stereospecific lignans [77].

In Melastomataceae, neither β-PGG nor tellimagrandin II have been isolated. However, nobotanin D (**30**), pterocaryanin C (**31**), casuarictin (**32**), and pedunculagin (**32a**) have been isolated from *M. multiflorum* and all Melastomataceous plants previously investigated (see Figure 7 and Table S7 in the Supplementary File). This means that the sequence of biaryl formation in this family starts in an early stage, 1,2,3,6-tetra-*O*-galloyl-β-d-glucopyranose (**29c**), found in *T. semidecandra* (Mart. and Schrank ex DC.) Cogn. The oxidative coupling between 2,3-*O*-galloyl groups first produces nobotanin D (**30**) and the subsequent galloylation on *O*-4 of **30** leads to pterocaryanin C (**31**), which produces casuarictin (**32**) by dehydrogenation of 4,6-*O*-galloyl groups as shown in Figure 7. These three monomers can be considered as the initial precursors in the biogenesis of nobotanins and melastoflorins isolated from Melastomataceae as shown in the Figure 9 and Figure 13.

The monomeric precursors should undergo two types of enzymatic degradations. One is the degalloylation catalyzed by a plant tannase such as that isolated from *Quercus ruber* [78]. This reaction might explain the biosynthesis of 2,3-*O*-(*S*)-HHDP-glucose (**32d**) from nobotanin D (**30**), pedunculagin (**32a**) from casuarictin, and praecoxin B (**31b**) from pterocaryanin C (**31**). The other is the de-hexahydroxydiphenoylation (de-HHDP-ation), which could be catalyzed by a hypothetic HHDP acyl hydrolase and give account of the formation of l,4,6-tri-*O*-galloyl-β-d-glucopyranose (**31a**) from pterocayanin C (**31**), along with strictinin (**32b**) and isostrictinin (**32c**) from casuarictin (**32**). These seven tannins are all monomeric units found in nobotanins and melastoflorins and will be regarded as pterocaryanin-type (**31b**, **31a**, and **31**) (Figure 13) and casuarictin-type (**32b**, **32**, **32a**, **32c**) (Figure 14) in the next section.

In *P. rotundifolia* (G. Forst.) Hook, three ellagitannins were identified as 6-*O*-galloyl-2,3-*O*-(*S*)-hexahydroxydiphenoyl-d-glucose (**30a**) [28,71], which could be derived by 1-*O*-degalloylation from nobotanin D (**30**), praecoxin B (**31b**) [28,79], and pterocarinin C (**31**) [7,28,42,71,80]. These ellagitannins yielded a product ion at *m*/*z* 301, representing the fragment ion of ellagic acid (**21**) [42].

The number of galloyl groups in these types of monomeric ellagitannis is evidenced by singlet signals integrating for two hydrogen atoms at 6.9–7.1 ppm in their ^1^H-NMR spectra, and each HHDP moiety is depicted as a pair of sp^2^ singlets signals between 6 and 6.8 ppm. Anomeric protons are represented by a doublet signal at 5.8–6.2 ppm with a wide coupling constant (*J* = 7–9 Hz) for the beta isomer, while the anomeric mix of those 1-OH free ellagitannins shows two doublet signals at δ 5–5.5 ppm, with *J* = 7–9 Hz for the beta anomer and *J* = 3–4 Hz for alpha anomers. The separation of enantiotropic H-6 proton signals is an indicator of pterocaryanin-type (∆δ H-6 about 0.3 ppm) or casuarictin-type (∆δ H-6 about 1.3 ppm) with a 4-O/6-O macrocycle [30] (see Table 1 and Table 2). The other aliphatic signals (H-2 to H-5) can be assigned on the basis of ^1^H-^1^H COSY, ^1^H-^1^H TOCSY, *J*-resolv, HSQC, and HMBC spectra. The (*S*)-configuration of both HHDP are evidenced from a strong positive Cotton effect ([θ] + 1.63 × 10^5^) at 235 nm in the CD spectrum [81].

**Table 1:** ^1^H-NMR of sugar moieties of pterocaryanin C-type monomeric ellagitannins.

*n*	Pterocaryanin C-Type			
	Pterocaryanin C (31)	Praecoxin B (31b) (α/β)	1,4,6-tri-*O*-Galloylglycopyranose (31a)	Nobotanin D (30)
	^1^H-NMR			
H-1	6.19 d (8.0)	5.48 d (3.5)/5.17 d (8)	5.80 d (8.5)	6.17 d (8)
H-2	5.14 dd (8.0, 1.0)	5.07 dd (9.5, 3.5)/4.88 dd (8, 9.5)	3.71 dd (8.0, 9.0)	5.05 dd (8, 10)
H-3	5.44 t (10)	5.62 t (9.5)/5.35 t (9.5)	3.96 t (9.0)	5.24 t (10)
H-4	5.58 t (10)	5.50 t (10)/5.46 t (9.5)	5.24 t (9.0)	3.98 t (10)
H-5	4.17 dd (3.0, 1.0)	4.54 ddd (10, 4, 2)/4.22 ddd (9.5, 5, 2)	4.14 m	4.07 m
H_a_-6/H_b_-6	4.46 br d (13)/4.24 dd (13.0, 13)	4.49 dd (12, 2)/4.27 dd (12, 4)/4.26 d (12, 5)	4.47 dd (14.0, 3.5) 4.14 m	4.60 dd (1.5, 12)/4.45 dd (5, 12)
Galloyl	7.16, 7.15, 7.13 (s)	7.142/7.139/7.13/7.10	6.92, 6.95, 7.02 (s)	7.12, 7.11, 2H (s)
2,3-HHDP	β1,4,6 (*S*)	4,6 (*S*)/6.60/6.59, 6.39		6.70, 6.42, β1,6 (*S*)
Ref.	[28,42,80,82,83,84]	[79]	[51,84]	[28]

**Table 2:** ^1^H-NMR of sugar moieties of casuarictin-type monomeric ellagitannins.

*n*	Casuarictin-Type			
	Casuarictin (32)	Pedunculagin (32a) (α/β)	Strictinin (32b)	Isostrictinin (32c)
	^1^H-NMR			
H-1	6.21 d (8.5)	5.43 d (3, 5)/5.22 d (9)	5.76 d (7)	6.08 d (8.5)
H-2	5.0 dd (8, 9)	4.83 dd (8.5, 9.5)	3.66 dd (7, 9)	4.99 dd (8.5, 9.0)
H-3	5.49 t (9.0)	5.44 t (10)/5.20 t (10.0)	3.84 t (9)	5.17 t (9.5)
H-4	4.99 t (10)		4.91 t (9)	3.88 t (9.5)
H-5	4.58 dd (5.7, 9)	4.18 ddd (10.0, 6.5, 1.5)/4.57 ddd (10.0, 7.0, 1.5)	4.11 dd (6, 9)	3.71 ddd (9.5, 5.5, 2.0)
H_a_-6/H_b_-6	3.84 d (13)/5.12 dd (6.5, 13)	5.25 dd (13.0, 6.5)/5.22 dd (13.0, 7.0)/3.82 dd (13.0, 1.5)/3.76 dd (13.0, 1.5)	5.22 dd (6, 14)/3.79 d (14)	3.87 d (12)/3.78 dd (12, 5.5)
Galloyl	7.18 (s) 2-H	----	7.16 (s) 2-H	7.12 (s) 2-H
2,3-HHDP	6.68, 6.55, β1 S	6.66/6.64, 6.60/6.59, S		6.70, 6.41
4,6-HHDP	6.47, 6.38, β1 S	6.55/6.50, 6.33/6.32, S	6.72, 6.61, β1 S	
Ref.	[22]	[85,86]	[84,85]	[84,85]

The anomeric carbons of monomeric ellagitannins show ^13^C-NMR signals remarkably downfield shifted to δ 90–100 ppm. In the anomeric mixtures like pedunculagin (**32a**) or praecoxin B (**31b**), two signals are observed with intensities indicating the relative α/β anomer concentrations. The C-6 signal could be evidence by a singlet at δ 60–64 ppm (see Table 3 and Table 4) and the other signals C-2 to C-5 are assigned on the basis of HSQC and HMBC [87,88].

**Table 3:** ^13^C-NMR and MS data of sugar moieties of pterocaryanin C type monomeric ellagitannins.

n	Pterocaryanin C-Type			
	Pterocaryanin C (31)	Praecoxin B (31b) (α/β)	1,4,6-Tri-*O*-galloylglycopyranose (31a)	Nobotanin D (30)
	^13^C-NMR			
C-1	91.9	91.3/94.9	95.4	92.1
C-2	75.3	75.1/77.7	73.7	79.9
C-3	77.4	75.4/77.6	75.2	76.2
C-4	67.8	68.7/68.5	71.5	75.3
C-5	73.9	68.4/73.1	73.7	67.8
C-6	62.7	63.1/63.2	63.3	63.6
ESI-MS	937 [M − H]^−^	785 [M − H]^−^	635 [M − H]^−^	786 [M − H]^−^
Ref.	[28,42,80,82]	[28,42,82,89,90]	[42,90]	[28]

**Table 4:** ^13^C-NMR and MS data of sugar moieties of casuarictin-type monomeric ellagitannins.

*n*	Casuarictin-Type			
	Casuarictin (32)	Pedunculagin (32a) (α/β)	Strictinin (32b)	Isostrictinin (32c)
	^13^C-NMR			
C-1	92.4	91.8	95.9	92.9
C-2	76.0	75.6	74.7	71.1
C-3	77.3	75.8	75.6	72.9
C-4	69.3	69.9	72.8	73.4
C-5	73.5	73.4	73.2	69.2
C-6	63.1	63.8	63.7	65.9
ESI-MS	935 [M − H]^−^	783 [M − H]^−^	633(100) [M − H]^−^	633(100) [M − H]^−^
Ref.	[22,64,82,84,91,92]	[82,84,92]	[64,82,84,90,92]	[64,82,84,92]

**Figure 17:**
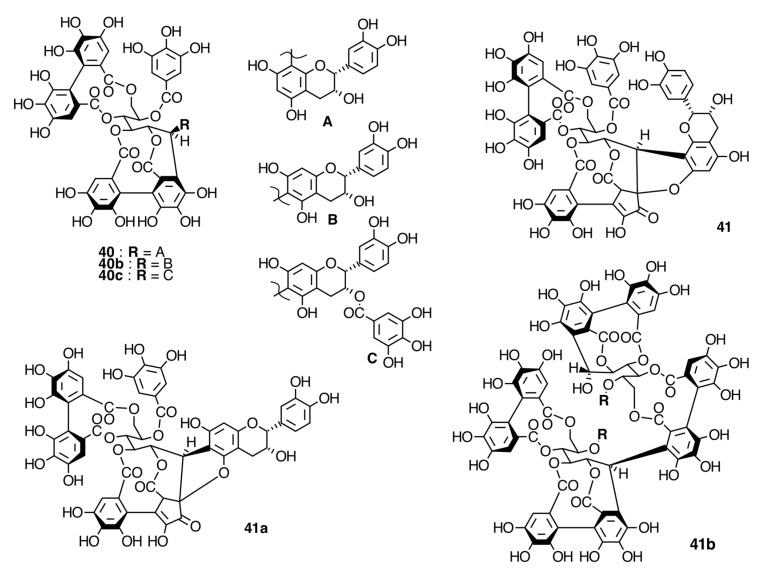
Complex tannins isolated in Melastomataceae species.

Among the representative ellagitannins are (*S*)-hexahydroxydiphenoyl (**33a**) esters of glucose with ^4^*C*_1_ conformation and (*R*)-HHDP (**33b**) or its derivative dehydrohexahydroxydiphenoyl (**33c**) esters with ^1^*C*_4_ glucopyranose (Figure 16). These may be two branches in an early stage of the biosynthetic pathway of monomeric hydrolyzable tannins in Melastomataceae. Additionally, *C*-glycosidic (Figure 12), complex tannins, (Figure 17) and oligomeric hydrolyzable tannins have also been isolated from Melastomataceous plants.

##### Oligomeric Ellagitannins

The *m*-DOG-type valoneoyl unit (**33d**) is probably the most often encountered inter-unit linkage in ellagitannin oligomerization via oxidative coupling processes [93]. The construction of the highest oligomers fully characterized to date such as the tetramer trapanin B (**33d**) [27], isolated from *Trapa japonica* Flerov (Trapaceae), and the pentamers melastoflorins A–D [28], isolated from *Monochaetum multiflorum* (Bonpl.) Naudin, relies on multiple occurrences of this unit type [94] (see Figure 8, Figure 9 and Figure 13).

The oligomeric ellagitannin dimers, trimers, and tetramers are classified into the *m*-DOG type [93,95], and they are characterized by the presence of the valoneoyl unit (**33d**) in the molecule. An alternate coupling among pterocaryanin C (**31**) and casuarictin (**32**) or their corresponding derivatives seems to be the common feature to the oligomerization in Melastomataceae species. Tannins isolated from *M. multiflorum* (Bonpl.) Naudin, including the new pentamers, also share the same feature [96].

**Figure 18:**
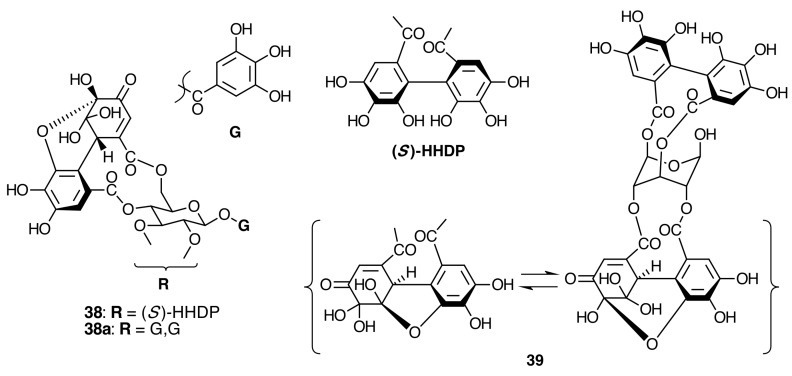
Ellagitannins with modified HHDP moieties.

The 1-butanol extract obtained from the aqueous acetone homogenate of the leaves of *Heterocentron roseum* A. Br. et Bouch., which is a shrub native to Mexico, yielded three new dimers, nobotanins G (**43a**), H (**43d**) and its lactone, andnobotanin I (**43g**) (depside form), upon fractionation and chromatography on Diaion HP-20 and Toyopearl HW-40. These dimers were structurally related to nobotanin B (**43**). Five known tannins, casuarictin (**32**), strictinin (**32b**), geraniin (**39**) (Figure 18), and nobotanins B (**43**) (the main tannin, 0.015% of the fresh leaves) and F (**42**) [97], were also isolated.

A possibility of artifacts found from the degradation of bigger oligomers during the isolation process for compounds **34**, **43d**, **43f**, and **43g** was ruled out by thermal requirements observed for partial hydrolysis of ellagitannins.

*M. malabathricum* Linn., a tree growing in Madagascar, India, and Malaysia, was also reported to produce three new dimers, named malabathrins B (**43e**), C (**43c**), and D (**43f**), along with 11 known hydrolyzable tannins [29]. The latter include monomers strictinin (**32b**), casuarictin (**32**), pedunculagin (**32a**), nobotanin D (**30**), and pterocaryanin C (**31**) along with dimers nobotanin B (**43**), G (**43a**), H (**43d**), and the trimer nobotanin J (**45**) [29]. A distinguishable feature of this plant was to produce complex tannins such as stenophyllanins A (**41**) and B (**41a**), and malabathrins A (**40**), E (**40a**), and F (**40b**), as well as *C*-glycosidic ellagitannins, casuarinin (**36**) and alienanin B (**41b**) [98] (see Figure 17 and Figure 18 and Tables S8 and S9 in the Supplementary File).

The purification of these tannins by chromatography on solid stationary phases was unsuccessful owing to degradation at the ester linkage of a valoneoyl unit on the anomeric center of the lower sugar unit. However, they were successfully purified by centrifugal partition chromatography, which enables quick development in the absence of a solid support. A subsequent study of a water soluble fraction obtained by liquid-liquid partition of 70% aqueous acetone homogenate from *Tibouchina semidecandra* (Schrank and Mart. ex DC.) revealed the occurrence of three new hydrolyzable tannins, nobotanins L (**44b**), M (**44c**), and N (**44d**), along with nobotanin C (**44a**) [99]. These tannins exist in solution as mixtures of α- and β-anomers. The anomeric proton signal, which up-field shifted near to δ 5.7 ppm, observed in the lower sugar units of 56 and 57, showed evidence of the un-acylation on O-2/O-3.

The 80% aqueous acetone extract from the whole plant of *Melastoma dodecandrum* Lour. yielded three monomers, casuarinin (**36**), casuarictin (**32**), and pedunculagin (**32a**), along with the dimer nobotanin B (**43**). These tannins were reported as inhibitors of NO production from activated macrophages treated with lipopolysaccharide and interferon-γ [31].

*C*-Glycosidic ellagitannins have also been isolated from *Bredia tuberculata* (Guillaumin) Diels and include the monomers casuarinin (**36**), castalagin (**37**), and stachyurin (**36a**), along with 1,3-di-*O*-galloyl-4,6-*O*-(*S*)-hexahydroxydiphenoylglucopyranose (**38**), pedunculagin (**32a**), and casuarictin (**32**), as well as dimeric nobotanins A (**42a**) and F (**42**), a new monomer, brediatin A(**35**), and a new dimer, brediatin B (**42b**), and nobotanins B (**43**), G (**43a**) and E (**44**). Similarly, *Melastoma normale* D. Don yielded the *C*-glycosidic monomeric ellagitannins casuarinin (**36**) and casuariin (**36b**) along with pedunculagin (**32a**), casuarictin (**32**), strictinin (**32b**), pterocaryanin C (**31**), and nobotanins B (**43**), G (**43a**), and H (**43d**) [30].

The structures of trimeric nobotanin J (**45**) and tetrameric nobotanin K (**46**), isolated from the 1-butanol extract of *Heterocentron roseum* Braun and Bouché, were completely characterized in 1995 [80].

The tannin composition of *Tibouchina multiflora* Cogn., collected in Colombia, was shown to be similar to that of *T. semidecandra* (Schrank and Mart. ex DC.). The 1-butanol extract afforded a new dimer, nobotanin O (**43b**), and a new tetramer, nobotanin P (**46a**), together with eight known compounds, stachyurin (**36a**), casuarinin (**36**), medillinin B (**42d**), nobotannins A (**42a**), B (**43**), C (**44a**), G (**46**), and J (**45**). Five known compounds, pedunculagin (**32a**), casuarictin (**32**), nobotanins D (**30**), F (**42**), and M (**44c**) [100], were isolated from the EtOAc extract.

As shown in Tables S8–S10 in the supplementary file, Melastomataceae species have been scarcely investigated and only a few species belonging to the three tribes Melastomeae, Miconieae and Sonerileae have been extensively studied. Furthermore, most of the reported natural products are polar compounds obtained from ethanolic extracts or aqueous acetone homogenates. Thus, considering the big size of the Melastomataceae, extensive research on natural products from members of this family still remains to be done.

In spite of the above circumstances, some generalizations can be attempted toward the chemotaxonomic relationships of the family. Out of hundreds of monomeric hydrolyzable tannins [93], only 27 have been found in Melastomataceae. Among these, the most frequently encountered tannins include casuarictin (**32**), pedunculagin (**32a**), nobotanin D (**30**), and pterocaryanin C (**31**), along with 1,4,6-tri-*O*-galloyl-β-d-glucopyranose (**31a**) and the *C*­glucosidic monomer casuarinin (**36**). They can be considered to be chemotaxonomical markers at the familiar level on the basis of a correlation at an early biogenetic stage. The absence of telimagrandin II, the isomer of **31**, is noticeable, which implies a characteristic order in the biosynthetic pathway of ellagitannins in Melastomataceae.

At tribal, generic, and specific levels, oligomeric hydrolyzable tannins, presumably formed at a late stage of biosynthesis, appear to be better taxonomic markers than monomers [95]. However, the available information is not enough to establish a chemotaxonomic key for the Melastomataceae species based on oligomeric ellagitannins. The same thing occurs regarding flavonoids and anthocyanins.

##### Biogenesis of Oligomeric Ellagitannins in Melastomataceae

The phytochemical analysis of *M. multiflorum* enriched the number of tetrameric ellagitannins found in Melastomataceae up to five and contributed the first pentameric hydrolyzable tannins, melastoflorins A–D, for this family. The results of this research provided additional evidence that nobotanin oligomers and new pentamers melastoflorins, composed of monomeric units with an alternate combination of casuarictin (**32**) and pterocaryanin C (**31**) (or their analogs), are characteristic of Melastomataceae. The structural similarity of nobotanins and melastoflorins led the proposal of a hypothetical biogenetic pathway map for these kinds of ellagitannins.

##### Biogenesis of Dimeric Hydrolyzable Tannins

The biogenesis of dimeric hydrolyzable tannins isolated from Melastomataceae has been proposed as the result of C-O oxidative coupling between one of 4-hydroxyl groups of a (*S*)-HHDP from a casuarictin-type monomeric unit and C-2 of the galloyl on C-4 of a pterocaryanin-type unit [71]. These monomers could be coupled in two manners. The first is conducting the formation of a valoneoyl group with the orientation *m*­DOG (4,6-4), as in nobotanin F (**42**) and its congeners nobotanins A (**42a**), R (**42c**), and brediatin B (**42b**), which will denominate route F (Figure 10). The second one is route B (Figure 11), which produces a valoneoyl group with *m*-DOG (3,2-4) as in nobotanins B, G (**43a**), and O (**43b**), as well as in malabathrins B (**43e**) and C (**43c**). In both biosynthetic routes, the C-O coupling may be proposed to be an enzymatically directed free radical process, although no enzymes have been described yet.

By these biogenetic routes, up to 18 dimeric ellagitannins might be biosynthesized, but only half have been isolated. Nobotanins H (**43d**) and I (**43g**) are exceptions of these routes and should be explained as the products from bigger ellagitannis bearing a *m*-DOG(2,3-l)-oriented valoneoyl group, which is degraded by a tannase-like enzyme.

##### Biogenesis of Trimeric Hydrolyzable Tannins

Trimeric ellagitannins could be produced by any of two paths, from routes F or B. The first one, route E (Figure 18), would produce nobotanins E (**44**), C (**44a**), L (**44b**), M (**44c**), and N (**44d**) by C-O coupling between the 2,3-HHDP group on casuarictin-type units of **79**, **78**, and **80** (route F) and the 4-*O*-galloyl group of other pterocaryanin C-type monomer. Thus, the new valoneoyl group has the orientation *m*-DOG (2,3-4).

**Figure 19:**
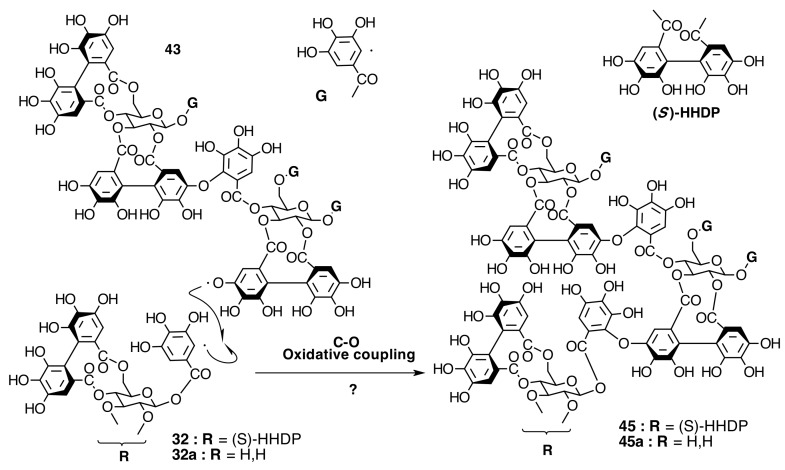
Route J in the biogenesis of trimeric ellagitannins in Melastomataceae. ? = Enzymes and reaction conditions are not yet clarified.

The other route, J (Figure 19), leads to the biogenesis of nobotanins J (**45**) and V (**45a**) by C-O coupling between the lower HHDP core of nobotanin B (**43**) and the galloyl group of a pterocaryanin C-type monomer. In this case, the new valoneoyl group is *m*-DOG (3,2-l)-oriented.

Although many compounds can be expected to be biosynthesized by routes E and J, only seven trimeric hydrolyzable tannins have been isolated in Melastomataceae. The 10th ellagitannin trimer, nobotanin U, should be formed in the same way as dimers **43d** and **43g**.

##### Biogenesis of Tetrameric Hydrolyzable Tannins

**Figure 20:**
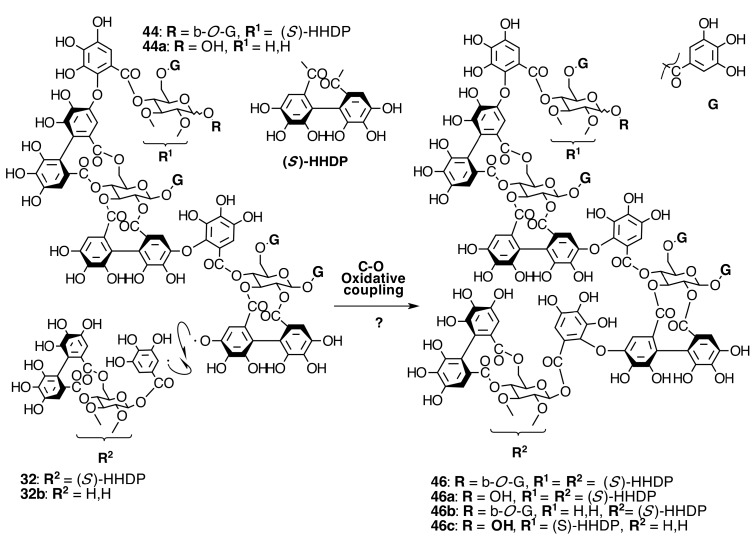
Route K in the biogenesis of tetrameric ellagitannins in Melastomataceae. ? = Enzymes and reaction conditions are not yet clarified.

The structural similarity of nobotanins C (**44a**), E (**44**), K (**46**), P (**46a**), Q (**46b**), and T (**46c**) suggests that they are biosynthesized by a common pathway. First, nobotanins E (**44**) and C (**44a**), both trimers [28], may be biosynthesized and, then, their 2,3 HHDP of glucose I undergoes C-O oxidative coupling with the anomeric galloyl group of an additional molecule of casuarictin (**32**) or strictinin (**32b**) in route K (Figure 20).

**Figure 21:**
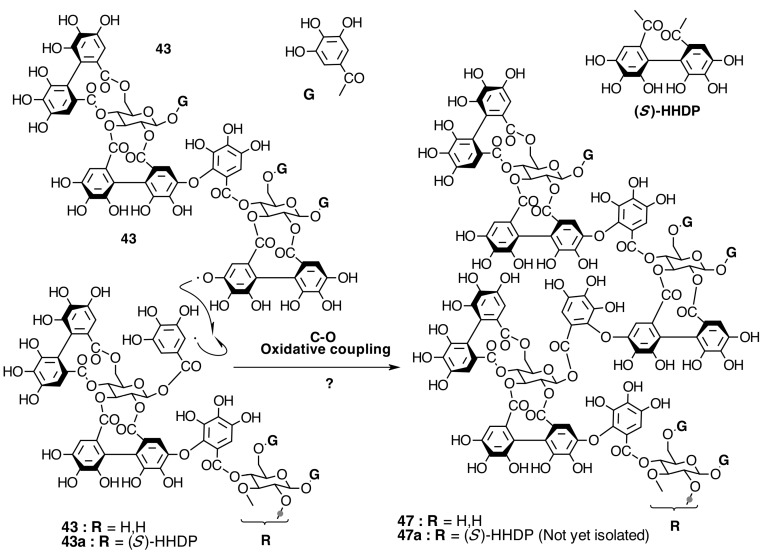
Route S in the biogenesis of tetrameric ellagitannins in Melastomataceae. ? = Enzymes and reaction conditions are not yet clarified.

However, the structure of nobotanin S (**47**), which is structurally related to melastoflorins, may be derived from the alternative biosynthetic route S (Figure 21). In this case, two stable dimers, nobotanins B and G, are synthesized first and then may be subjected to C-O oxidative coupling, just as described above to produce **47**. The involved enzyme in route S should be different from that in routes J (Figure 19) and K (Figure 20).

##### Biogenesis of Melastoflorins A–E

**Figure 22:**
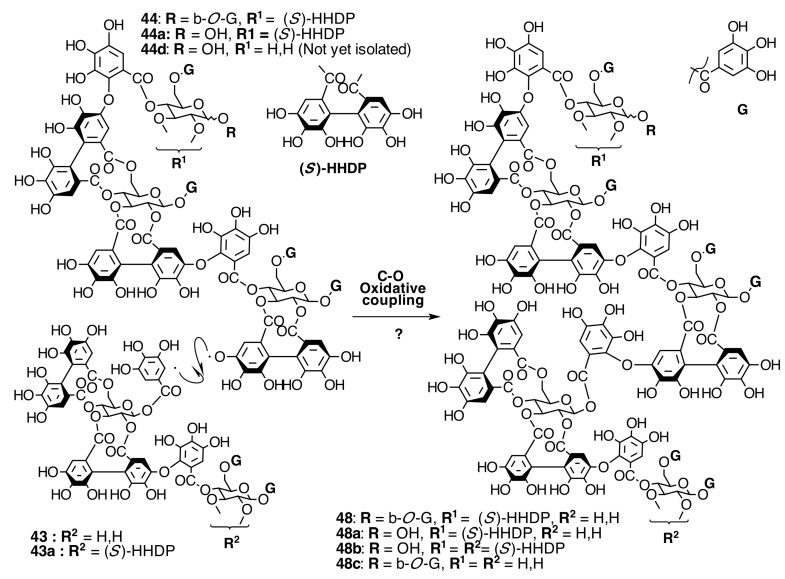
Route ES in the biogenesis of pentameric ellagitannins in Melastomataceae. ? = Enzymes and reaction conditions are not yet clarified.

Melastoflorins A (**48**), B (**48a**), C (**48b**), and D (**48c**), isolated from *Monochaetum multiflorum* (Bonpl.) Naudin, would be proposed to be biosynthesized by route ES (Figure 22). The C-O oxidative coupling between the 2,3-HHDP group on glucose I of nobotanins E (**44**), C (**44a**), and the hypothetical **44d** and the 1-*O*-galloyl group on glucose II of nobotanins B (**43**) or G (**43a**) produces the corresponding melastoflorins [96]. The possible biogenesis of melastoflorins A–D from nobotanin S (**47**) and a pterocaryanin C-type monomer cannot be ruled out. Finally, the biogenetic route ES (Figure 22) opens the door to look for hexameric hydrolyzable tannins, which might be produced by C-O oxidative coupling between two trimers from routes E (Figure 16) or J (Figure 19) or three dimers from routes F (Figure 9) and/or B (Figure 10).

### 2.7. Condensed Tannins

This type of polyphenol has not been isolated or characterized from Melastomataceous plants; however, phytochemical assays of *Bellucia dichotoma* Cogn. showed that it contains flavonoids, terpenoids, and condensed and hydrolyzable tannins [101]. The colored reaction of 70% aqueous acetone of *Miconia prasina*, *M. trinervia*, *M. coronata*, *B. glossularioides*, *B. pentamera*, and *Aciotis purpuracens* leaves with HCl/*n*-Butanol gave positive results for condensed tannins in a test tube [47].

## 3. Conclusions

Despite the large number of secondary metabolites of phenolic and polyphenolic types isolated from Melastomataceae plants, the number of species studied is too low compared to the size of the family, and furthermore, only three of the nine tribes have been investigated. These facts motivate additional investigations of Melatostomataceus plant species to isolate and characterize those condensed tannins detected in some of them. From the biosynthetic viewpoint, it is important to isolate and characterize the enzymes involved in the metabolic pathways proposed in this review. This will be important to promote biotechnological developments that will facilitate the production and isolation of phenols and polyphenols of this plant family.

## Supplementary Materials

Supplementary materials can be accessed at: http://www.mdpi.com/1420-3049/20/10/17818/s1.
