# Supplementary material for: Phenolics and Polyphenolics from Melastomataceae Species
Source: Molecules. 2015 Sep 25;20(10):17818–47. doi: 10.3390/molecules201017818 (PMC6332314; doi:10.3390/molecules201017818)
Supplement: Supplementary file 1 [file molecules-20-17818-s001.pdf]

Supplementary Materials

1. Taxonomy of Melastomataceae

Melastomataceae, the seventh largest family of flowering plants, belong to the order myrtales along with aristolochiaceae, combretaceae, crypteroniaceae, halorrhagidaceae, lythraceae, memecylaceae, myrtaceae, onagraceae and rhizophoraceae.

Table S1. Summary of Melastomataceae taxonomy (Adapted from Renner, 1993 [2]).

Subfamilies	Tribes
Melastomatoideae (Naudin 1849)	Astronieae (Triana, 1865) 4 Genera, 149 species
	Blackeeae (Hook,1867) 2 Genera, 162 species
	Microlicieae (Naudin, 1849) 11 Genera, 67 species
	Rhexieae (D.C 1828) 1 Genus, 13 species
	Sonerileae (Triana, 1865) 40 Genera, 560–600 species
	Miconieae (D.C 1828) 38 Genera, 2200 species
	Merianieae (Triana, 1865) 16 Genera, 220 species
	Melastomeae (Osbeckeae, D.C 1828) 47 Genera, 850 species
Kibesioideae(Naudin, 1849)	Kibessieae (Krasser, 1893) Pternandra (15 spp.)

Jussieu (1789) first recognized the melastomataceae as a natural unit; however, David Don (1823) was who put structure into the family. Triana, a Colombian native with extensive knowledge in the field, published his system in 1865 and slightly modified it in 1871. Triana's system grouped the melastomataceae in three subfamilies, melastomatoideae, astronioideae and memecyloideae; which include thirteen tribes. Owing to the size of this family, the internal classification has been reviewed several times. A recent systematic analysis of melastomataceous plants re-structured and placed them into two subfamilies, kibesioideae and melastomatoideae, which contains only nine tribes (see Table S1) and separated memecyloideae and crypteronioideae to upgrade them to the family status, memecylaceae and crypteroniaceae respectively [2].

2. Folk Medicinal Uses of Melastomataceous Plants

Melastomataceous plants have been long used as traditional medicines, especially in Asia and Latin America. In Indonesia, Malaysia and China, some species of this family, such as *Melastoma*, *Medinilla* and *Osbeckia* species, have been used as remedies for diarrhea, dysentery, leukorrhea, and various skin diseases, as well as astringents or hemostatics [8]. The tannins and the presence of alkylated derivatives of ellagic acid in various plants of this group may be responsible for these medicinal applications [9]. Various phytochemical studies have revealed ellagitannins as the main constituents.

In China, a decoction of the leaves of *Melastoma dodecandrum* Lour. is suggested to be effective as a wash for scrofula, hemorrhoids, dermatitis, leprosy, scabies, athlete's foot, and snakebites, as well as to prevent abortion. The root juice is taken as a remedy for post parturient abdominal pains and dysentery [8]. The whole plant has been used as a traditional antipyretic, antitoxic, diuretic, hemostatic and anti-rheumatic medicine. An 80% aqueous acetone extract of the whole plant exhibited anti-allergic activity in the passive cutaneous anaphylaxis test and toward histamine release from rat peritoneal mast cells with an antigen antibody reaction *in vitro* [10]. The leaves of *Melastoma malabathricum* Linn.,

which is known as "daunharendong" in Indonesia and Malaysia, have been used as a crude drug for the treatment of diarrhea, dysentery and leucorrhea [11–13].

In Chiapas Mexico, people eat the leaves of *Arthrostema ciliatum* Pavón ex Don. In another parts of Central America, the decoction of the whole plant is used as diuretic, purgative and as refreshment [14]. A syrup prepared from flowers of *Rhychanthera grandiflora* (Aubl.) DC. has been cited as a natural medicine against respiratory illnesses in French Guyana [15].

Arthrostema volubile (Bompl. Ex Naudin) Triana and *A. macrodesmum* Gleason are used in Colombia in decoction of their stems or whole aerial part of the plant as a febrifuge, especially in the treatment of malaria. The stems are chewed or in the form of sap to quench the thirst and to cure the gum irritations. The decoction of the whole plant of *Brachyotum strigosum* (L.f.) Triana is used against the calculus of the bladder and other ailments of genitourinary tract; whereas the decoction of *Chaetolepis microphylla* (Bompl.) Miq. is used as diuretic [16].

Bellucia dichotoma Cogn. is one of various plant species used in folk medicine in the west of the state of Pará, Brazil, to treat snake bites. The extract inhibits the local effects of *Bothrops atroxvenomas* as suggested by the inhibition of phospholipase A2, hemorrhagic and edematogenic activities [17].

In Colombia, some species of the family have been used as folk medicine for the treatment of malaria, infections, skin injuries, respiratory diseases, bladder calculus and other genitourinary ailments, as diuretic and as a topical remedy for gum irritations. Verbal information submitted by settlers from Caldas, Colombia indicates that *Tibouchina ciliaris* Vent.) Cogn. and *Monochaetum multiflorum* (Bonpl.) Naudin are traditionally used for treatment of skin infections and injuries.

A recent and concise review on melatomataceous plants medicinal uses has been published [18], and another review correlating the chemical structure and gastric anti-ulcer activity of tannins [19].

Table S2. Triterpenoids and alkyl benzoquinones in melastomatacea species.

Tribes	Species	Compounds			
		Triterpenoids	***Alkyl Benzoquinones***	***Acyl Glycosides***	**Ref.**
1	*Tibouchina candolleana*	(**1a**); (**2a**)	(**1**); (**2**); (**3**)		[12]
2	*Miconia stenostachya*	(**1b**); (**1c**)			[9]
	Miconia lepidota		(**4**); (**4a**)		[10]
3	*Centrademia grandiflora*	(**1b**)			[9]
	Dissotis perkinsiae	(**1a**); (**2a**)		(**3a**)	[11]

Tribes: 1. Melastomeae, 2. Miconieae, 3. Sonerileae. **Compounds**: (**1**): β-amirin; (**1a**): Oleanolic acid; (**1b**): Sumaresinolic acid; (**1c**): 3-*epi*-sumaresinolic acid; (**2**): α-amirin; (**2a**): Ursolic acid; (**3**): β-sitosterol; (**3a**): sitosterol 3-*O*-β-D-glucopyranoside; (**4**): 2-Methoxy-6-pentyl-3,5-cyclohexadiene-1,4-dione; (**4a**): 2-Methoxy-6-heptyl-3,5-cyclohexadiene-1,4-dione.

Table S3. Flavonoids, flavonoid glycosides and acyl glycosides reported for melastomataceous plants.

Tribes	Species	Compounds			Ref.
		Flavonoids	*Flavonoids Glycosides*	*Flavonoids Acyl Glycosides*	
1	*Monochaetum multiflorum*		(**6c**); (**8c**); (**8d**)	(**8h**); (**8i**)	[15,16,19]
	Melastoma malabathricum	(**5**)		(**8f**)	[11,29]
	Melastoma normale		(**9a**)		[30]
	Melastoma dodecandrum	(**5**)	(**5c**)		[31]
	Tibouchina candolleana	(**7**)		(**8f**); (**8g**)	[12]
	Tibouchina semidecandra	(**8**); (**9**); (**10**); (**10a**)	(**8a**); (**8c**); (**8e**); (**8l**)		[28]
2	*Miconia cabucu*	(**5**); (**8**); (**9**)	(**6f**); (**8b**); (**8c**); (**8k**); (**9a**); (**9b**); (**9c**) (**14**)		[26]
	Miconia rubiginosa	(**9**); (**11**); (**12**); (**12a**)	(**9a**); (**9b**); (**9c**)		[22]
	Miconia prasina		(**13a**); (**13b**); (**13c**); (**13d**); (**13e**)		[24]
	Medinilla magnifica	(**6**); (**8**); (**10**); (**10a**)			[13]
	Clidemia floribunda	(**6**)			[13]
	Clidemia rubra	(**11**); (**12**); (**12a**)		(**9a**); (**9b**); (**9c**)	[23]
3	*Bertolonia marmorata*	(**8**); (**10**); (**10a**)			[13,14]
	Centrademia grandiflora			(**8h**); (**8i**)	[13,25]
	Centrademia floribunda	(**6**)		(**8f**); (**8g**)	[13]
4	*Huberia glazioviana*	(**5**); (**6**)	(**5a**); (**5b**)	(**8c**); (**8d**); (**8e**)	[20]
	Huberia minor	(**5**)	(**6b**); (**6c**); (**8c**); (**8d**); (**8e**)		[21]
	Huberia nettoana	(**5**)	(**6c**); (**8c**); (**8d**); (**8e**)		[21]
	Huberia ovalifolia	(**5**)	(**5b**); (**6f**); (**8c**); (**8d**); (**8e**)		[21]
	Huberia semiserrata	(**5**); (**6**)	(**6b**); (**6d**); (**6e**); (**8c**); (**8e**)		[21]
	Huberia carvalhoi	(**5**)	(**5a**); (**5c**); (**8e**)		[21]
	Huberia consimilis	(**5**)	(**5a**)		[21]

Tribes: 1. Melastomeae, 2. Miconieae, 3. Sonerileae, 4. Merianeae. **Compounds**: (**5**): Apigenin; (**6**): Kaempferol; (**7**): Luteolin; (**8**): Quercetin; (**9**): Myricetin; (**10**): Leucocyanidin; (**10a**): Leucodelphinidin; (**11**): Catechin; (**12**): Epicatechin; (**12a**): Epigallocatechin gallate; (**5a**): Apigenin 7-*O*-glucoside; (**5b**): Apigenin 7-*O*-(Glucose-rhamnose); (**5c**): Vitexin; (**6b**): Kaempherol-7-*O*-glucoside; (**6c**): Trifolin; (**6d**): Kaempherol-7-*O*-(galactose-xylose); (**6e**): Kaempherol-7-*O*-(glucose-rhamnose); (**6f**): Kaempferol-3-*O*-β-D-(6″-coumaroyl)- glucopyranoside; (**8a**): Avicularin; (**8b**): Quercetin 3-*O*-α-L-(2″-*O*-acetyl)-arabinofuranoside; (**8c**): Hyperin; (**8d**): Isoquercitrin; (**8e**): Quercitrin; (**8f**): Isoquercitrin 6″-*O*-gallate; (**8g**): Hyperin 6″-*O*-gallate; (**8h**): Hyperin 6″-*O*-caffeate; (**8i**): Isoquercitrin 6″-*O*-caffeate; (**8k**): Quercetin-3-*O*-α-L-rhamnopyranosyl-(2-1)-*O*-β-D-xylopyranoside; (**8l**): Quercetin 3-*O*-α-L-rhamnopyranoside; (**9a**): Myricitrin, (**9b**): Myricetin 3-*O*-glucoside; (**9c**): Myricetin 3-*O*-rhamnosyl-glucoside; (**13a**): Matteucinol; (**13b**): Farrerol; (**13c**): Desmethoxymatteucinol; (**13d**): Miconioside C; (**13e**): Miconioside B; (**14**): Hydroxy-4′,7-dimethoxyflavone-(6-C-6″)-5″-hydroxy-3‴,4‴,7″-trimethoxyflavone.

Table S4. Anthocyanins isolated from melastomataceous plants.

Tribes	Species	Compounds		Ref.
		Anthocyanins	*Malvidin Acyl Glycosides*	
1	*Melastoma malabathricum*	(**15**); (**16**); (**17**); (**18**); (**19**)	(**19a**); (**19b**); (**19c**); (**19d**)	[27,33,34]
	Tibouchina grandiflora		(**17a**); (**17b**); (**19a**); (**19b**); (**19h**)	[36]
	Tibouchina granulosa		(**19d**); (**19e**); (**19f**); (**19h**)	[35]
	Tibouchina urvilleana		(**19g**)	[37]
	Tibouchina semidecandra	(**15**); (**16**); (**17**); (**18**); (**19**)	(**19a**); (**19b**); (**19c**)	[27,33,34]
	Tibouchina multiflora	(**19g**)		[36]
2	*Clidemia hirta*		(**16a**); (**16b**); (**18a**); (**18b**); (**18c**); (**19c**); (**19e**); (**19f**)	[23]
3	*Dissotis perkinsiae*			

Tribes: 1. Melastomeae, 2. Miconieae, 3. Sonerileae. **Compounds**: (**15**): Pelargonidin; (**16**): Cyanidin; (**17**): Peonidin; (**18**): Delphinidin; (**19**): Malvidin; (**16a**): Cyanidin 3-*O*-glucoside; (**16b**): Cyanidin 3-*O*-rutinoside, (**17a**): Peonidin-3-sophoroside; (**17b**): Peonidin-3-sambubioside; (**18a**): Delphinidin 3-*O*-diglucoside; (**18b**): Delphinidin 3-*O*-rutinoside; (**18c**): Delphinidin 3,5-*O*-diglucoside; (**19a**): Malvidin-3-*O*-glucoside; (**19b**): Malvin; (**19c**): Tibouchinin; (**19d**): Malvidin-2-*p*-coumaroylxylosyl-5-glucoside; (**19e**): Malvidin-3-*p*-coumaroylxylosyl-5-glucoside; (**19f**): Malvidin-3-(di-*p*-coumaroylxylosyl)-5-glucoside; (**19g**): Malvidin 3-*O*-[6-*O*-(*E*)-*p*-coumaroyl-β-D-glucopyranosyl]-5-*O*-(2-*O*-acetyl-β-D-xylopyranosyl); (**19h**): Malvidin-3-*p*-(coumaroylsambubiosyl)-5-glucoside.

Table S5. Phenol acids and derivatives from melatomataceae species.

Tribes	Species	Compounds	Ref.
		Phenol Acids and Derivatives	
1	*Monochaetum multiflorum*	(**20**); (**20a**); (**20b**); (**21**); (**21a**); (**22**); (**23**); (**24**)	[19]
	Tibouchina semidecandra	(**21a**)	[32]
2	*Phylagathis rotundifolia*	(**21b**); (**21c**)	[42]
	Clidemia floribunda	(**23**)	[41]
3	*Dissotis perkinsiae*	(**21**)	[11]

Tribes: 1. Melastomeae, 2. Miconieae, 3. Sonerileae. **Compounds**: (**20**): Protocatechuic acid; (**21**): Ellagic acid; (**20a**): Gallic acid; (**20b**): Methyl gallate; (**21a**): 3,3′-*O*-dimethyl ellagic acid 4-*O*-α-L-rhamnopyranoside; (**21b**): 3,3′,4-tri-*O*-methylellagic acid 4′-*O*-β-D-glucopyranoside; (**21c**): 3′-*O*-methyl-3,4-methylenedioxyellagic acid 4′-*O*-β-D-glucopyranoside; (**22**): Valoneic acid dilactone; (**23**): Caffeic acid; (**24**): 4-*O*-β-D-glucopyranosyl-2-*O*-methylphloroacetophenone.

Table S6. Galloylated cyanogenic glucosides from melatomataceae species.

Tribes	Species	Compounds	Ref.
		Galloylated Cyanogenic Glucosides	
1	*Monochaetum multiflorum*	(**26**); (**27**); (**28**)	[19,23,37]
2	*Phylagathis rotundifolia*	(**25**); (**25a**); (**25b**); (**25c**); (**25d**); (**25e**); (**25f**)	[19,42,44]

Tribes: 1. Melastomeae, 2. Miconieae. **Compounds**: (**25**): 6'-*O*-gallate prunasin; (**26**): Benzyl 6'-*O*-galloyl-β-D-glucopyranoside; (**27**): 4-*O*-(6'-*O*-galloyl-β-D-glucopyranosyl)-*cis*-*p*-coumaric acid; (**28**): Di-hyperin ester of tetrahydroxy-µ-truxinic acid (monochaetin); (**25a**): Prunasin 2′,6′-di-*O*-gallate; (**25b**): Prunasin 3′,6′-di-*O*-gallate; (**25c**): Prunasin 4′,6′-di-*O*-gallate, (**25d**): Prunasin 2′,3′,6′-tri-*O*-gallate; (**25e**): Prunasin 3′,4′,6′-tri-*O*-gallate; (**25f**): Prunasin 2′,3′,4′,6′-tetra-*O*-gallate.

Table S7. Galloyl glucoses and Monomeric ellagitannins reported for melastomataceae species.

Tribes	Species	Compounds		Ref.
		Galloyl Glucoses	*Monomeric Ellagitannins*	
1	*Monochaetum multiflorum*	(**20b**); (**29b**); (**29l**); (**31a**)	(**30**); (**31**); (**32**); (**32a**); (**32c**)	[19]
	Melastoma malabathtricum	(**29l**); (**31a**)	(**30**); (**31**); (**32**); (**32a**); (**32b**)	[29]
	Melastoma normale	(**29l**); (**31a**)	(**31**); (**32**); (**32a**); (**32b**)	[30]
	Melastoma dodecandrum	(**20c**)	(**32**); (**32a**)	[31]
	Tibouchina semidecandra	(**29b**); (**29c**); (**31a**)	(**30**); (**31b**); (**32**); (**32a**); (**32d**); (**34**)	[71]
	Tibouchina multiflora		(**30**)	[42]
	Heterocentrum roseum		(**32**); (**32b**)	[29]
2	*Medillinia magnifica*		(**34a**)	[71]
	Phylagathis rotundifolia	(**29d**); (**29h**); (**29i**); (**29j**); (**29k**)		[48,53]
3	*Bredia tuberculata*		(**32a**); (**35**)	[30]

Tribes: 1. Melastomeae, 2. Miconieae, 3. Sonerileae. **Compounds**: (**20b**): Benzyl 6′-*O*-galloyl-β-D-glucopyranoside; (**20c**): Gallic acid 3-*O*-(6-*O*-galloyl)-β-D-glucopyranoside; (**29b**): 1,2,6-tri-*O*-galloyl-β-D-glucopyranose; (**29c**): 1,2,3,6-tetra-*O*-galloyl-β-glucopyranose; (**29d**): 1,2,3,4,6-penta-*O*-galloyl-β-D-glucose; (**29h**): 6-*O*-galloyl-D-glucose; (**29i**): 3,6-di-*O*-galloyl-D-glucose; (**29j**): 1,2,3-tri-*O*-galloyl-β-D-glucose; (**29k**): 3,4,6-tri-*O*-galloyl-D-glucose; (**29l**): 1,2,4,6-tetra-*O*-galloyl-β-D-glucopyranose; (**31a**): 1,4,6-tri-*O*-galloyl-β-D-glucopyranose; (**30**): Nobotanin D; (**31**): Pterocaryanin C; (**31b**): Praecoxin B; (**32**): Casuarictin; (**32a**): Pedunculagin; (**32b**): Strictinin; (**32c**): Isostrictinin; (**32d**): 2,3-*O*-(*S*)-HHDP-D-glucopyranose; (**34**): Praecoxin A; (**35**): 1,3-di-*O*-galloyl-4,6-*O*-(*S*)-HHDP-β-D-glucopyranose; (**34a**): Medillinin A.

Table S8. *C*-Glucosidic monomeric ellagitannins reported for melastomataceae species.

Tribes	Species	Compounds			Ref.
		C-Glucosidic Monomeric Ellagitannins	*Complex Tannins*	*Dehydroellagitannins (Monomers)*	
1	*Melastoma dodecandrum*	(**36**)			[31]
	Melastoma normale	(**36**); (**36b**)			[30]
	Melastoma malabathrichum		(**40**); (**40a**); (**41**); (**41a**)		[79]
	Hetrocentron roseum			(**39**)	[80]
	Tibouchina semidecandra	(**36**)			[71]
	Tibouchina multiflora	(**36**); (**36a**); (**37**); (**37a**); (**37b**)			[91]
2	*Bredia tuberculata*	(**37**)		(**38**)	[30]

Tribe: 1. Melastomeae, 2. Sonerileae. **Compounds**: (**36**): Causarinin; (**36a**): Stachyrin; (**36b**): Casuariin; (**37**): Castalagin; (**37a**): Vescalagin; (**37b**): Methylvestalagin; (**38**): 1,3-di-*O*-galloyl-4,6-*O*-(*S*)-hexahydroxydiphenoylglucopyranose; (**39**): Geraniin; (**40**): malabathrin A; (**40a**): malabathrin E; (**41**): stenophyllanin A; (**41a**): stenophyllanin B.

Table S9. Dimeric and Dimeric *C*-glucosidic ellagitannins from melastomataceae species.

Tribes	Species	Compounds		Ref.
		Dimeric Ellagitannins	***Dimeric C-Glucosidic Ellagitannins***	
1	*Monochaetum multiflorum*	(**42**); (**42a**); (**42b**); (**42c**); (**43**); (**43b**)		[30]
	Melastoma malabathrichum	(**43**); (**43a**); (**43c**); (**43d**); (**43e**); (**43f**)	(**41b**)	[92]
	Hetrocentron roseum	(**42**); (**43**); (**43a**); (**43g**)		[80]
	Tibouchina semidecandra	(**42**); (**42a**); (**43**);		[61]
	Tibouchina multiflora	(**42**); (**42a**); (**42d**) (**43**); (**43a**); (**43b**)		[98]
	Medinilla magnifica	(**42d**)		[30]
3	*Bredia tuberculata*	(**42**); (**42a**); (**42b**); (**43**); (**43a**)		[30]

Tribes: 1. Melastomeae, 3. Sonerileae. **Compounds**: (**42**): Nobotanin F; (**42a**): Nobotanin A; (**42b**): Brediatin B; (**42c**): Nobotanin R: (**42d**): Medillinin B; (**43**): Nobotanin B; (**43a**): Nobotanin G; (**43b**): Nobotanin O; (**43c**): Malabathrin C; (**43d**): Nobotanin H; (**43e**): Malabathrin B; (**43f**): Malabathrin D; (**43g**): Nobotanin I; (**41b**): Alienanin B.

Table S10. Trimeric, tetrameric and pentameric ellagitannins ellagitannins from melastomataceae species.

Tribes	Species	Compounds			Ref.
		Trimeric Ellagitannins	***Tetrameric Ellagitannins***	***Pentameric Ellagitannins***	
1	*Monochaetum multiflorum*	(**44**); (**45**); (**45a**); (**46**); (**46b**); (**46c**); (**47**)		(**48**); (**48a**); (**48b**); (**48c**)	[80]
	Melastoma malabathrichum	(**45**)			[98]
	Hetrocentron roseum	(**45**)	(**46**)		[80]
	Tibouchina semidecandra	(**44**); (**44a**); (**44b**); (**44c**); (**44d**)			[80]
	Tibouchina multiflora	(**44a**); (**44c**); (**45**)	(**46a**)		[92]
2	*Bredia tuberculata*	(**44**)			[98]

Tribes: 1. Melastomeae, 2. Sonerileae. **Compounds**: (**44**): Nobotanin E; (**44a**): Nobotanin C; (**44b**): Nobotanin L; (**44c**): Nobotanin M; (**44d**): Nobotanin N; (**45**): Nobotanin J; (**45a**): Nobotanin V; (**46**): Nobotanin K; (**46a**): Nobotanin P; (**46b**): Nobotanin Q; (**46c**): Nobotanin T; (**47**): Nobotanin S; (**48**): Melastoflorin A; (**48a**): Melastoflorin B; (**48b**): Melastoflorin C; (**48c**): Melastoflorin D.
